# Analysis of rice nuclear-localized seed-expressed proteins and their database (RSNP-DB)

**DOI:** 10.1038/s41598-020-70713-8

**Published:** 2020-09-15

**Authors:** Priyanka Deveshwar, Shivam Sharma, Ankita Prusty, Neha Sinha, Sajad Majeed Zargar, Divya Karwal, Vishal Parashar, Sanjeev Singh, Akhilesh Kumar Tyagi

**Affiliations:** 1 Interdisciplinary Centre for Plant Genomics and Department of Plant Molecular Biology, University of Delhi, South Campus, New Delhi, India; 2 Proteomics Laboratory, Division of Plant Biotechnology, Sher-e-Kashmir University of Agricultural Sciences & Technology of Kashmir, Shalimar, Srinagar, Jammu & Kashmir India; 3 Institute of Informatics and Communications, University of Delhi, South Campus, New Delhi, India

**Keywords:** Molecular biology, Plant sciences

## Abstract

Nuclear proteins are primarily regulatory factors governing gene expression. Multiple factors determine the localization of a protein in the nucleus. An upright identification of nuclear proteins is way far from accuracy. We have attempted to combine information from subcellular prediction tools, experimental evidence, and nuclear proteome data to identify a reliable list of seed-expressed nuclear proteins in rice. Depending upon the number of prediction tools calling a protein nuclear, we could sort 19,441 seed expressed proteins into five categories. Of which, half of the seed-expressed proteins were called nuclear by at least one out of four prediction tools. Further, gene ontology (GO) enrichment and transcription factor composition analysis showed that 6116 seed-expressed proteins could be called nuclear with a greater assertion. Localization evidence from experimental data was available for 1360 proteins. Their analysis showed that a 92.04% accuracy of a nuclear call is valid for proteins predicted nuclear by at least three tools. Distribution of nuclear localization signals and nuclear export signals showed that the majority of category four members were nuclear resident proteins, whereas other categories have a low fraction of nuclear resident proteins and significantly higher constitution of shuttling proteins. We compiled all the above information for the seed-expressed genes in the form of a searchable database named Rice Seed Nuclear Protein DataBase (RSNP-DB) https://pmb.du.ac.in/rsnpdb. This information will be useful for comprehending the role of seed nuclear proteome in rice.

## Introduction

Proteins function in multiple ways in a cell, and each protein is destined to a particular cellular organelle for its function. After protein synthesis in the cytoplasm, the proteins are sorted to different compartments in the cell. The nucleus is a subcellular compartment that is DNA’s abode. Proteins present inside the nucleus have special functions. Most of them interact with DNA to regulate the processes of DNA replication and transcription. Nuclear proteins like transcription factors and chromatin remodeling proteins regulate the expression of the genes. Gene regulatory nature of nuclear proteins renders significance to their study. The mechanism of nuclear localization regulation is complex^1^. The best-understood mechanism of nuclear transport is karyopherin mediated^2,3^, which can be either importins or exportins. Importin α recognizes the classical nuclear localization signal (cNLS) on a protein and makes a complex with it and recruits importin β. Once the importin-cargo complex is assembled, importin β engages in the nuclear pore complex (NPC) and passes through it. In the nucleoplasm, binding of GTP bound RAs-related Nuclear protein (Ran-GTP) facilitates the unloading of the cargo from importins. For the export of a protein from the nucleus, it must carry a nuclear export signal (NES). The NES is recognized by the exportin that subsequently binds to the protein to be exported and to Ran-GTP. This complex is docked at the NPC and, thereafter, passes through it to the cytoplasm. The whole process of nuclear transport is energy-dependent and involves the conversion of Ran-GDP to Ran-GTP, wherein an asymmetric gradient of the two forms of Ran (Ran-GTP and Ran-GDP) is maintained between nucleus and cytoplasm^4^. However, the mere presence of an NLS does not guarantee nuclear localization. Many factors influence the nuclear targeting efficiency of a nuclear localization signal (NLS)^5^. Sometimes the NLS is masked and requires specific post-translational modifications that may be conditional^6^. Thus, the movement of many proteins into the nucleus is also provisional viz., dependent on developmental, stress level, or hormonal changes. Further, a protein may or may not spend its entire life in the nucleus. Conditional shuttling of a protein between the nucleus and other non-nuclear locations determines its functional elasticity. For example, phytochromes are well-known proteins that move in or out of the nucleus in response to light^7^. NLSs are also not very well defined. Many uncharacterized peptide sequences also have the abilities of an NLS^8^. Further, some proteins can act as carriers of other non-NLS containing proteins^9^. Thus, movement of a protein in the nucleus is dependent on many factors.

Few attempts have been made to understand the protein composition of the plant nucleus^10^. Studies using a proteomics approach to identify nuclear protein composition, involve the separation of nuclei followed by protein isolation. Quite a few nuclear proteome studies have been done using the vegetative tissues in plants like in *Chlamydomonas*^11^, *Arabidopsis*^12^, chickpea^13^ and rice^14–18^. However, only a few reports are available wherein seed nuclear proteomes are analyzed^19^. Seeds are abundant in storage components like starch, fats and storage proteins, which make the isolation of nuclear protein difficult; however, some protocols have been standardized to purify the nuclei and thereof the proteins from seed samples. In *Medicago truncatula*, nuclear proteome component corresponding to 143 proteins was identified from seeds at 12 days after pollination (DAP)^20^. In maize, different developmental stages of endosperm were used to isolate their nuclear proteomes and apparent differential protein composition was observed on a one dimensional (1D) gel^21^. In wheat, 528 proteins corresponding to nuclear proteome of seed developmental stages viz., endosperm cell division and cell differentiation were identified in two wheat species^22^. Also, 464 nuclear proteins were identified as differentially expressed in different stages of seed development in wheat^23^. In rice, 468 proteins were identified from the nuclear fraction of 9 DAP endosperm^24^.

Grain filling/seed development in rice is a ~ 30-day long process and genes expressed during this period govern the quality of the seed. The expression of most genes is regulated by transcription factors and other nuclear factors that interact with them. Transcriptome studies in both *Arabidopsis* and rice have shown upregulation of a number of transcription factors in the early stages of seed development^25,26^. The identification and understanding of nuclear proteins during seed development allow comprehending the module of gene regulatory network active in seed development.

Given the availability of rice expression data, we know the transcript abundance of genes in different stages of seed development. Thus, if we can predict the nuclear-localized proteins component of these seed-expressed genes without actually performing the localization experiments, one can define the seed nuclear proteome. This ability can be pivotal to pin down the candidates of gene regulatory nature in seed development for their functional characterization. Given the variability in the modes of nuclear localization of proteins, the nuclear prediction should be a compilation of many measures for nuclear localization. Most of the organellar localization prediction tools incorporate protein sequence information wherein pre-documented sorting signals and amino acid composition are used to predict the localization^27–32^. However, some tools can identify novel sorting signals for protein localization^33,34^. Other advanced prediction tools also utilize information of motifs, domains, homology in other species, GO and text-mining^35–37^. Hybrid approaches by combining multiple criteria to predict sub-cellular localization have shown better predictability^38–42^. We have attempted to identify the nuclear protein composition of rice seed development by combining the results of four online subcellular prediction tools, experimentally validated literature and nuclear proteome data. Moreover, we have compiled all the information about the seed-expressed genes and their localization in the cell in the form of a database called ‘Rice Seed Nuclear Protein DataBase’ (RSNP-DB). The data is complemented with the latest experimental evidence of the cellular localizations as realized from published literature. This database can act as a resource for prioritizing the molecular targets for the functional characterization of seed development regulatory components.

## Results

### Identification of seed-expressed nuclear-localized proteins

**Figure 1:**
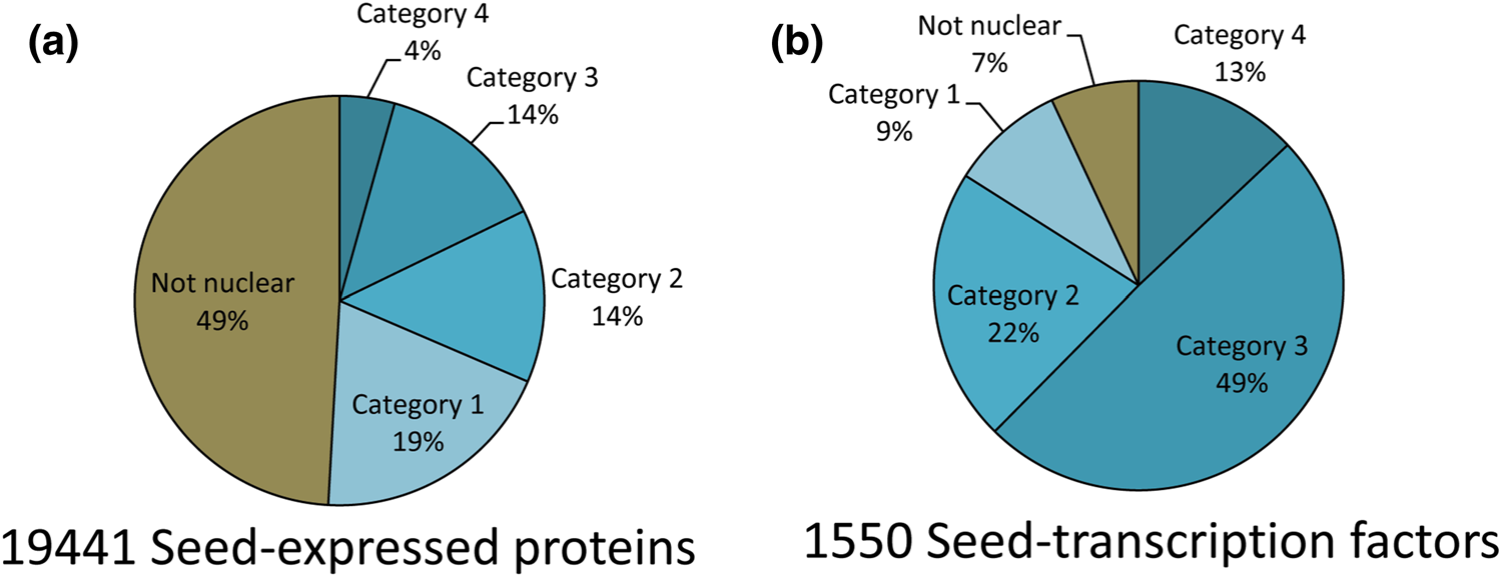
Nuclear prediction of proteins encoded by seed-expressed genes and transcription factors. (**a**) Pie chart showing the distribution of proteins of seed-expressed genes in different categories of nuclear localization. Proteins predicted to be nuclear localized by anyone, any two, any three and all four of the online subcellular localization tools used are indicated as categories 1, 2, 3 and 4, respectively. Those not predicted nuclear by any prediction tool are called not nuclear. (**b**) Pie chart showing the distribution of seed-expressed transcription factors in different categories of nuclear prediction.

We used our previous microarray data to identify genes expressed during seed development in rice^25,43^. Rice Genome Annotation Project (RGAP) locus IDs corresponding to 19,441 seed-expressed unique non-transposable element genes were identified. To ascertain their protein localization, we retrieved the encoded protein sequences of all these genes and analyzed them for their subcellular localization using four online subcellular localization prediction tools, namely, WoLF-PSORT^27,44^, YLoc^41^, CELLO^42^ and NucPred^34^. These four online tools used overlapping as well as variable criteria to predict the organellar localization of a protein. These criteria were based on the presence of sorting signals, the composition of amino acids, functional motifs and sequence similarity to proteins with known localization. Predictions by all the 4 tools are based on amino acid sequences and the presence of sorting signals. However, WoLF-PSORT is a more generic tool that identifies the classical NLSs and also considers the composition of the type of amino acids in the proteins. NucPred is a tool specific for the identification of nuclear proteins and is trained to discover new unknown NLSs in addition to the predefined ones. CELLO and YLoc are prediction tools based on hybrid prediction approaches. CELLO predictions are based on homology searches and sequence annotations, whereas YLoc also includes protein motifs and gene ontology (GO) terms for subcellular localization prediction. We made a cumulative list of the four predictions with their respective scores for all the 19,441 seed-expressed genes (Supplementary Table S1). This analysis revealed 50.8% proteins (9892 proteins) to be localized in the nucleus by at least one of the softwares, whereas an almost equal number of proteins (9549 proteins, 49.1%) were predicted as non-nuclear by all the four softwares. Subsets comprising of 3776, 2654, 2617 and 845 proteins were predicted nuclear by any one, any two, any three and all four softwares, respectively (Fig. 1a, Supplementary Table S1). These protein subsets are hereafter referred to as category 1, category 2, category 3 and category 4, respectively.

Next, we wanted to identify the robustness of calling a protein nuclear in these four categories. Transcription factors (TFs) are a major component of nuclear proteins with established roles in DNA binding and regulation. Thus, TF composition was identified in all the categories. Of the 9549 proteins called non-nuclear, only 1.13% (108) proteins were transcription factors, whereas 3.70% (140), 12.62% (335), 29.27% (766) and 23.78% (140) proteins of the category 1, 2, 3 and 4, respectively, were transcription factors (Fig. 1b, Supplementary Table S1). Thus a total of 93% of all transcription factors expressed (9%, 22%, 49%, 13% belonging to category 1, 2, 3and 4, respectively) during seed development were found in the subsets wherein at least one of the software is predicting the protein to be nuclear. Further, moving from category 1 to category 2 and above, a significant increase in the TF frequency was observed.

### Gene ontology analysis of seed-expressed nuclear-localized proteins

**Figure 2:**
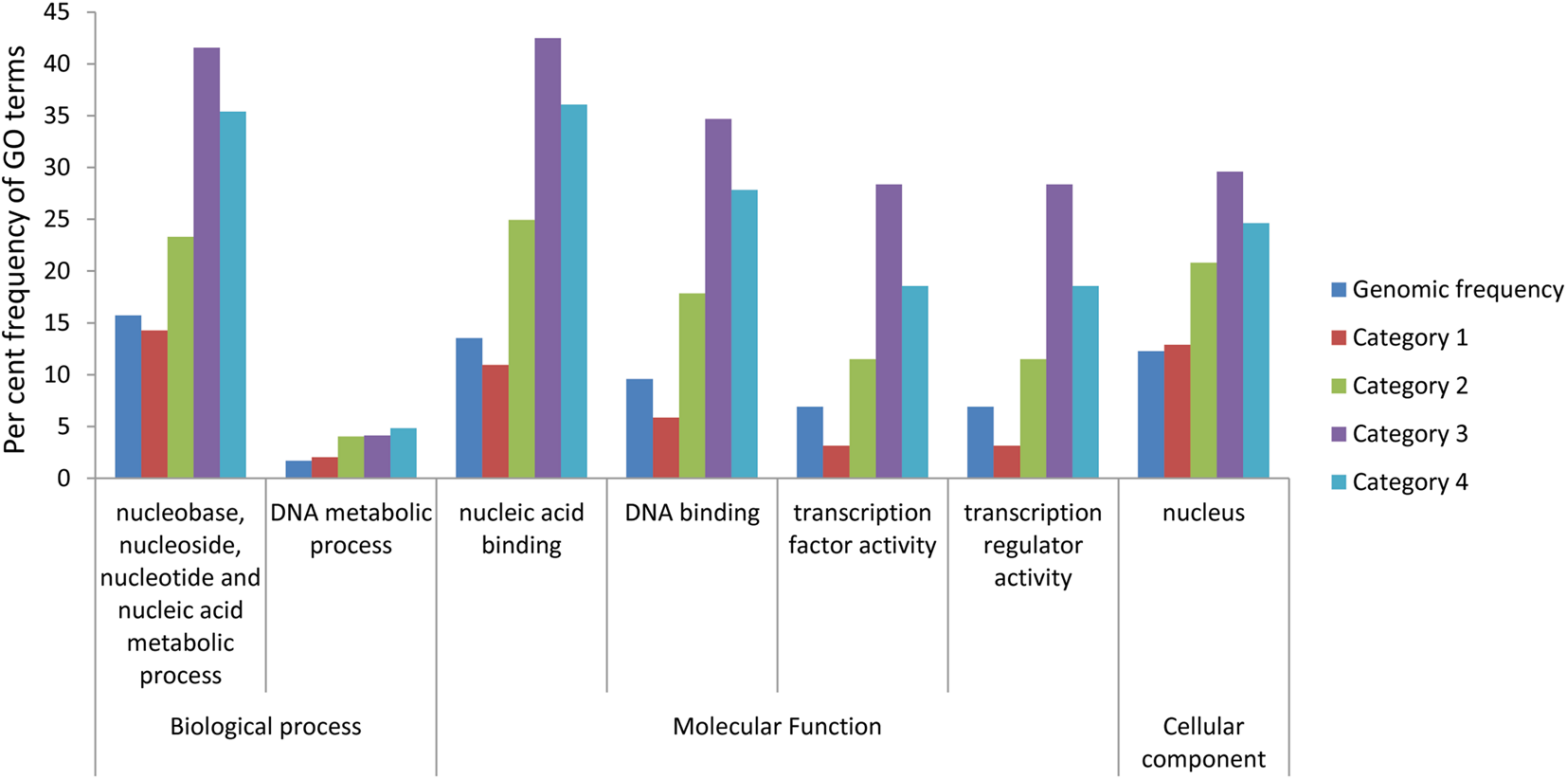
Distribution of nucleus related gene ontology terms in the different nuclear-predicted categories. Per cent frequency of two GO terms that describe the biological processes of the nucleus, four GO terms that describe the molecular functions in the nucleus and the GO term ‘nucleus’ under GO category ‘cellular component’ are shown for the four categories. The genomic reference is given for enrichment comparisons.

Gene ontology (GO) terms are a reflection of the kind of genes in a particular set of genes. GO analysis of seed-expressed genes showed that GO term ‘nucleus’ of ‘cellular component’ exhibited a 1.70, 2.41, 2.01-folds enrichment in categories 2, 3 and 4, respectively, in relation to the whole genome reference. Moreover, nuclear function related ‘biological process’ GO terms like ‘nucleobase, nucleoside, nucleotide and nucleic acid metabolic process’ and ‘DNA metabolic process’ showed enrichment of 1.48 and 2.35; 2.64 and 2.41; 2.25 and 2.82-folds, respectively, in category 2, 3 and 4. Molecular function GO terms that are relevant to nuclear function include, ‘nucleic acid-binding’, ‘DNA binding’, ‘transcription factor activity’ and ‘transcription regulator activity’. They also showed considerable enrichment (nucleic acid binding—1.84, 3.14, 2.66-folds; DNA binding—1.86, 3.61, 2.90-folds; transcription factor and regulator activity each—1.66, 4.10, 2.68-folds) in category 2, 3 and 4 respectively. Notably, enrichment of all nucleus related GO terms (except DNA metabolic process) was highest in category 3, followed by category 4. Also, all the above-stated terms were either under-represented or equivalent to the whole genome average frequency in category 1 (Fig. 2, Supplementary File 1). By combining the TF and GO analysis, it appears that nuclear localization prediction of proteins by category 2, 3, and 4 (6116 proteins) is more credible than category 1.

### Evidence from the literature and experimental validation support the prediction

**Figure 3:**
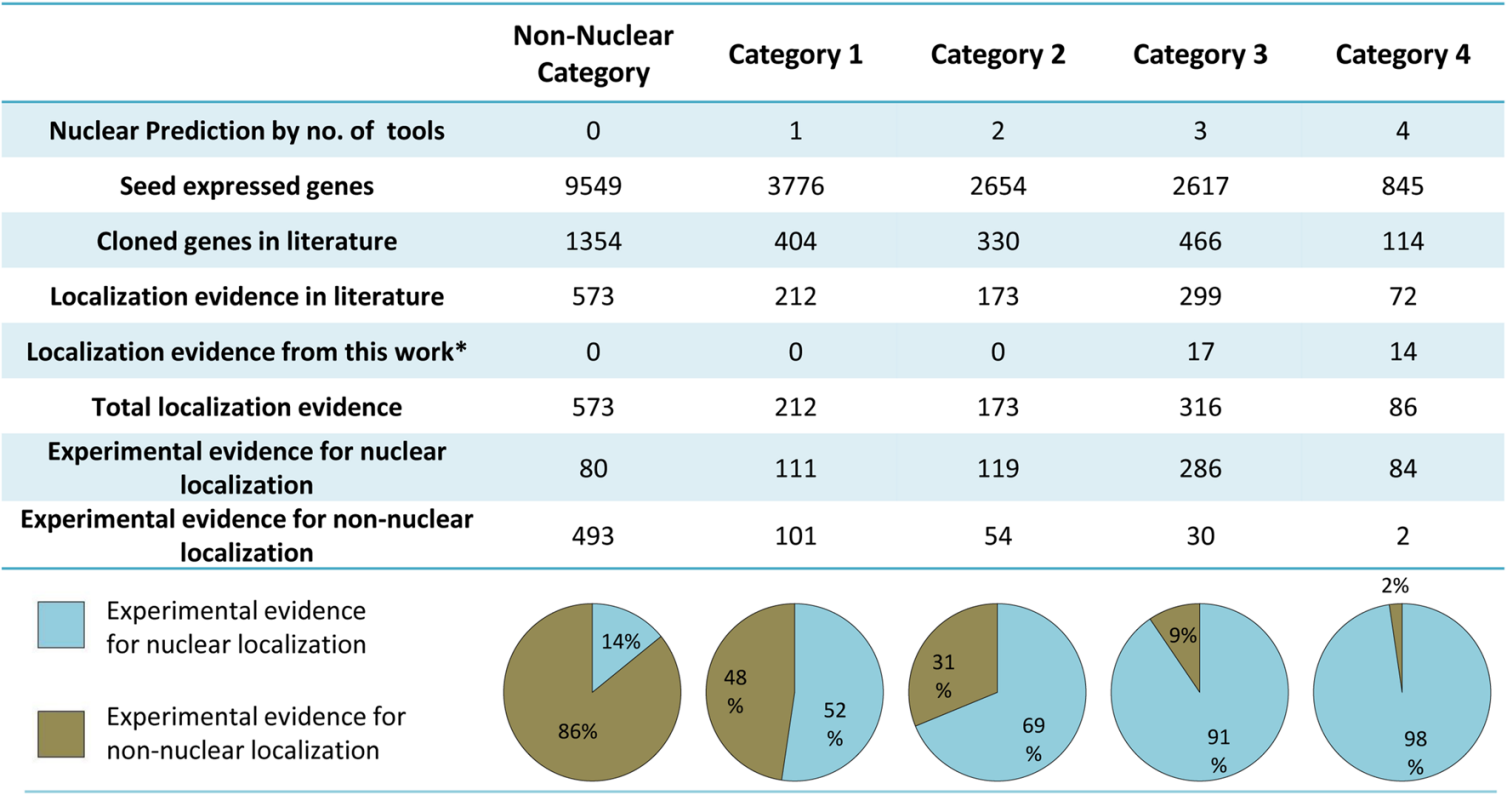
Experimental evidence for subcellular localization. The number of proteins in each category is indicated. The proportion of evidenced nuclear and non-nuclear proteins in each category is shown as a pie chart. *See Supplementary File 2 for localization of 31 proteins.

Further, we wanted to compare the robustness of the predictions with the experimental data. For this, we identified all rice genes that were cloned and were reported in any published literature. This list of genes was retrieved from funRiceGenes (https://funricegenes.github.io/)^45^. Of the 19,441 seed-expressed genes, 2668 genes were found to be cloned and published till 1st January 2020. These publications were checked individually to find whether protein localization data is available or not. Experimental evidence of the localization information was available for 1329 proteins (Fig. 3). To add to the literature-based evidence, we selected candidate genes from different categories and performed localization experiments. We cloned 31 proteins in fusion with YFP and observed their subcellular localization in tobacco leaf epidermal cells (Supplementary File 2). By combining the literature-based evidence and our localization results, we could catalog localization evidence for 1360 seed-expressed proteins. Noticeably, 97.67% and 90.51% of the proteins with localization evidence under category 4 and category 3 were nuclear (Fig. 3), whereas, the percentage of such nuclear localization evidenced protein reduced to 68.79% and 52.36% for category 2 and category 1, respectively. Enquiringly, 13.96% of the published protein under the non-nuclear category also showed nuclear localization. These results suggest that nuclear localization of categories 3 and 4 can be substantiated with more confidence indicated by evidenced data.

We used the experimental localization evidence supported data to calculate the accuracy of predictability of the 4 prediction tools. We found that out of the evidence supported 680 nuclear proteins, CELLO could accurately predict as high as 79.12% proteins to be nuclear, followed by YLoc (71.62%), WoLF-PSORT (60.44%) and NucPred (15.74%).

### Nuclear proteome studies consolidate the nuclear prediction

**Figure 4:**
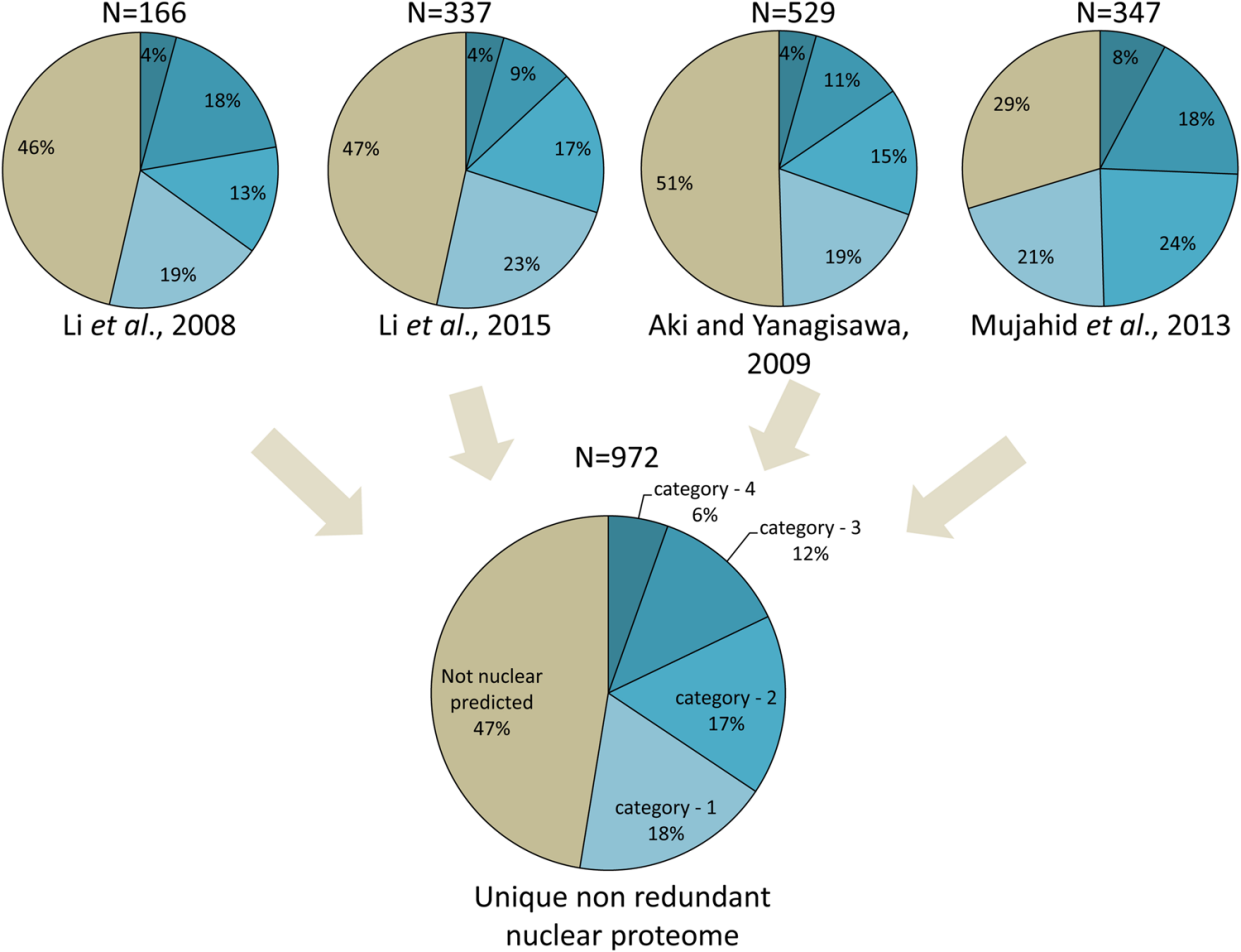
Comparison of the experimental nuclear proteome with nuclear prediction categories. Four nuclear proteome reports from rice involving protein isolation of nuclei followed by identification of the protein component were used to identify a subset of nuclear proteome of seed-expressed genes. Pie charts show the distribution of different nuclear prediction categories in the nuclear proteome component.

Another way of direct evidence of nuclear-localized proteins is the isolation of the nucleus and thereby its protein component followed by protein identification. In rice, we could identify four such “nuclear proteome” studies, wherein the universal accession IDs of the proteins were available. These proteomic studies included isolation of nuclei from endosperm^24^, embryo^46^, seedling^18^ and protoplast^47^. Though loci for 468, 362, 556 and 382 proteins were reported, respectively, as nuclear proteome of endosperm (9 days after pollination), embryos from imbibed seeds, seedling (5 weeks old) and protoplast, only 166, 337, 529 and 347 loci were found to be seed-expressed in our data. We compared these datasets with our different categories of nuclear proteins (Fig. 4). By combining all the nuclear proteome data, a non-redundant set of 1132 proteins is identified, wherein 972 are seed-expressed according to our data. The prediction tools can identify 52.57% as nuclear, whereas a little less than half the experimental nuclear proteome (47.42%) is not predicted as nuclear by any of the prediction tools (Fig. 4, Supplementary Table S2). Analysis of 972 nuclear proteome proteins showed that only 6.06% of them are TFs that are significantly less than TF composition found earlier by us in categories 1, 2, 3 and 4. Moreover, those TFs present in nuclear proteome data were majorly histones, histone binding factors and other TFs involved in epigenetic regulation (SET domain, HMG domain, BAH domain, Alfine-like domain, SWI domain, SNF2 domain-containing proteins). Large TF families (like MADS BOX, Homeobox, B3 domain, WRKY, AP2 domain, AUX/IAA, ARF) were either altogether absent or meagrely represented (1 BHLH, 1 NAC, 1 MYB, 1 NF-Y, 4 BZIP, 1 C2H2) in the nuclear proteome data. These results showed that proteins of higher abundance are picked up in proteome studies, but proteins with smaller concentrations but important functions are missed. Also, seed storage glutelin proteins and seed allergen proteins that are abundant in seed tissue were picked up in embryo and endosperm nuclear proteomes as contaminations. Ribosomal proteins were identified as the dominant category in nuclear proteome studies. We did a keyword search “ribosome” in the function category of 19,441 seed-expressed genes and found a list of 293 (1.51%) different kinds of ribosomal proteins. Similar searches were done for (i) 9892 gene loci that were nuclear predicted by category 1, 2 and 3; and (ii) 972 nuclear proteome identified loci. Only 147 genes (1.49%) matched the predicted nucleome (i), whereas 54.94% (161 proteins) were picked up in a much smaller number of nuclear proteome fraction (ii). Only 4.3% of the nuclear proteome identified ribosomal proteins were in category 3 or 4, with more than 50% called non-nuclear.

### Nuclear localization and export signals govern the protein localization

**Figure 5:**
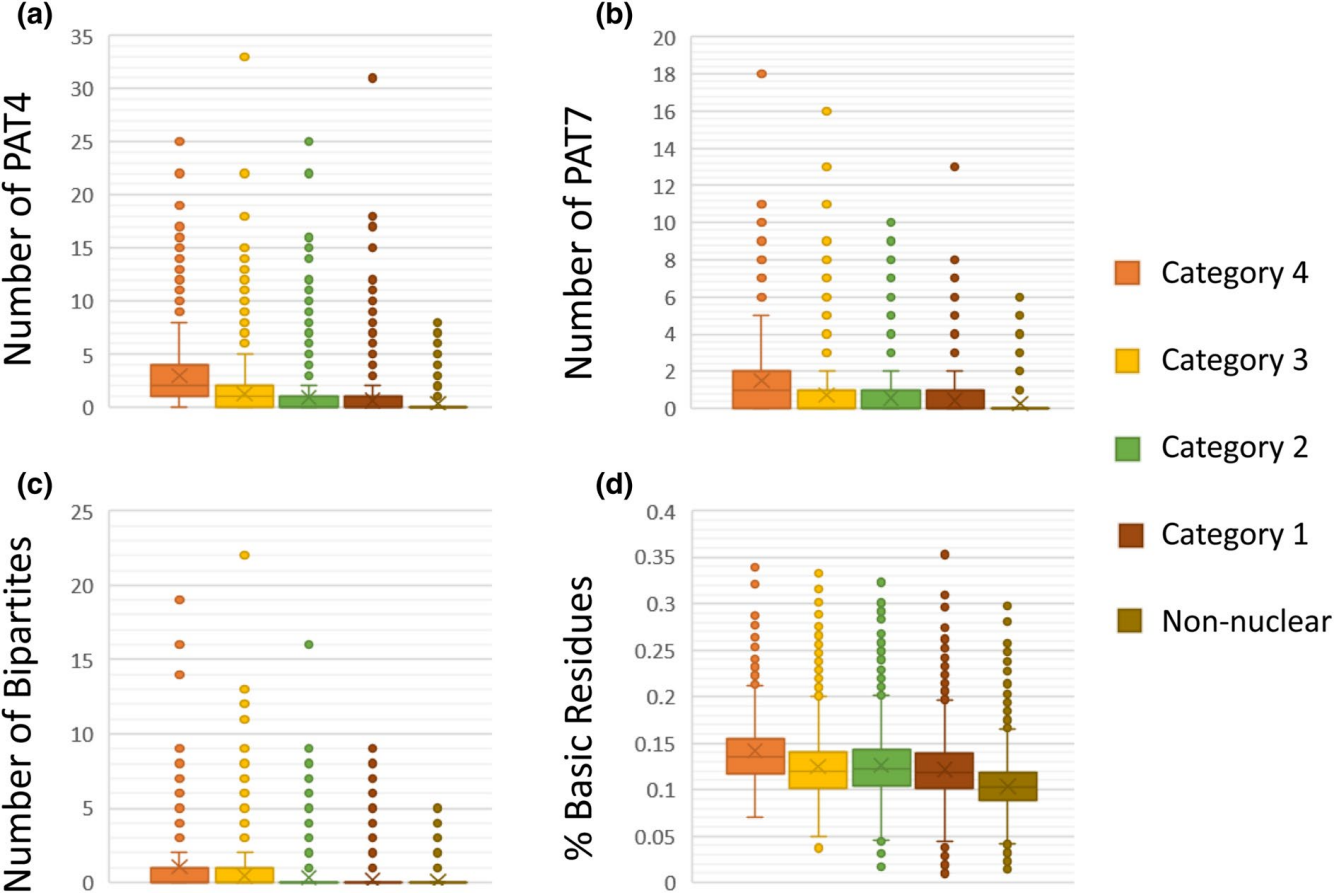
Protein sequence analysis of seed-expressed genes for the presence of nuclear localization signals (NLS) in different categories of nuclear-predicted protein subsets. Box whisker plots displaying the distribution of the number of (**a**) PAT4 sequences, (**b**) PAT7 sequences, and (**c**) bipartite sequences in the five categories of protein subsets. (**d**) Box plot showing variation in the percentage of basic residues in the protein sequences of five predicted categories. Outliers are shown as dots.

Most nuclear localization signals (NLSs) are short stretches of basic amino acids like lysine and arginine. Importin α/β mediated import of cargo to the nucleus is mediated by the recognition of NLS. Classical NLS are of two kinds, namely, monopartite and bipartite. Classical monopartite NLS is a single cluster of basic amino acids whereas classical bipartite NLS has a linker region between two groups of basic amino acids. Monopartite NLSs are majorly of two kinds, pattern 4 (PAT4) and pattern 7 (PAT7) with a length of 4 and 7 amino acids, respectively. We analyzed the presence of PAT4, PAT7, and bipartite NLS signals using PSORT II and calculated their frequency distribution in proteins from all categories. The distribution of number of NLS signals and basic residues in all the categories is shown in Fig. 5. A set of 87.69% of protein from category 4 showed the presence of at least 1 PAT4 signal. The subsequent reduction was observed in categories 3, 2 and 1, respectively, with 52.81%, 42.10% and 32.15% protein sequences with a minimum of one PAT4 signal (Fig. 5a, Supplementary Table S3). Likewise, proteins with at least one PAT7 signal had a composition of 65.91%, 42.14%, 34.28% and 27.17% correspondingly in category 4, 3, 2 and 1 (Fig. 5b, Supplementary Table S3). Bipartite signals also showed higher enrichment in categories 3 and 4 (Fig. 5c). A minimum of one bipartite signal was seen in 48.6% and 26.82% proteins of the category 4 and 3, respectively, whereas only 18.88% and 12.71% proteins of category 2 and 1 possessed any bipartite signal (Supplementary Table S3). Per cent basic amino acid composition of respective categories showed decreasing constituent levels of arginine and lysine in the proteins (Fig. 5d, Supplementary Table S3).

**Figure 6:**
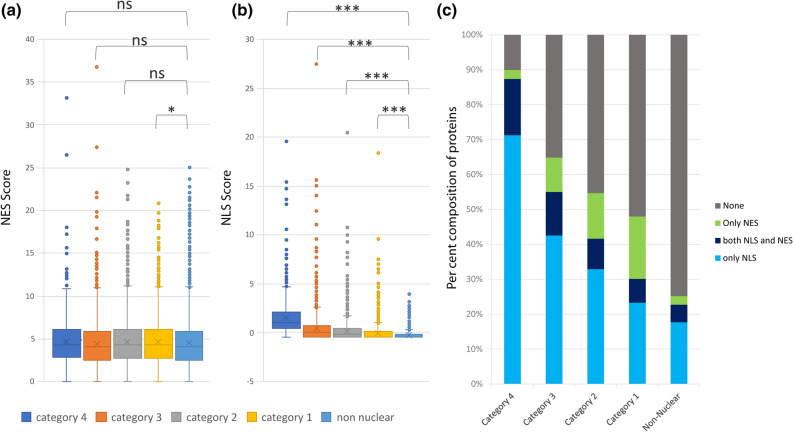
Presence of nuclear localization signal (NLS) and nuclear export signal (NES) in the protein sequences of genes corresponding to categories 1, 2, 3, 4, and non-nuclear. Box whisker plots showing the distribution of NES scores (**a**) and NLS scores (**b**) amongst the proteins of the five categories. (**c**) Per cent distribution of the proteins containing NLS, NES, and co-existence of both signals is shown. ***Represents p-value of the t-test less than 0.0001, *represents p-value of the t-test less than 0.01, *ns* not significant p-value.

Nuclear export signal (NES) is present in most of the nuclear proteins that shuttle between the nucleus and elsewhere. We identified the NES signals in all the seed-expressed genes using NetNES 1.1 Server (https://www.cbs.dtu.dk/services/NetNES/)^48^. On the basis of NES score given to each amino acid of the protein, a cumulative NES score was calculated for each protein. The distribution analysis of NES scores does not show any significant difference between different categories (Fig. 6a). Likewise, the cumulative NLS score was determined for each protein based on different NLS determining criteria shown in Fig. 5 using PSORT II. The NLS score distribution, however, showed significantly higher scores in the proteins of category 4 compared to the non-nuclear category (Fig. 6b). Further, the NLS and NES scores were converted into a definite presence or absence call of NLS and NES in the protein, respectively. Protein sequences containing at least three NES contributing amino acid residues within a stretch of 15 amino acids were designated as NES containing proteins. Likewise, a positive NLS score was considered as NLS containing protein. We could identify 4567 and 6193 proteins with an NES and NLS, respectively, in seed-expressed genes. We compared the co-existence of NLS and NES in the protein sequences of seed-expressed genes (Supplementary Table S4). Figure 6 summarizes the per cent contributions of the two kinds of signals (NES and NLS) in the five categories. The fraction of NLS containing proteins was highest in category 4 (87.34%), which decreased to 54.99%, 41.60%, 30.11% and 22.70% in subsequent categories. However, NES containing proteins were present in more or less same fractions in categories 1, 2 and 3 with 24.68%, 21.78% and 22.32% proteins, respectively, whereas category 4 proteins showed a lesser number (18.69%) of NES containing proteins. Non-nuclear category showed only 7.48% proteins carrying NES (Fig. 6c). The fraction of proteins having only NES and no NLS was highest in category 1 (17.85%), followed by category 2 (13.07%). Protein types wherein both NLS and NES were present were maximum in category 4 (16.09%), followed by category 3 (12.45%). These observations indicated that category 4 majorly constitutes nuclear resident proteins, whereas categories 3, 2 and 1 have more numbers of shuttling proteins with both NLS and NES or only NES. These results can also explain that experimental evidence for nuclear proteins belonging to category 4 was majorly restricted to the nuclear compartment with minuscule or no signal in non-nuclear locations. Out of the 84 proteins with evidence of nuclear-localization of category 4, as many as 78.57% (66 proteins) showed nucleus only localizations. On the other hand, proteins from categories 3, 2, and 1 showed lesser exclusive nuclear compartmentalization (68.88%, 61.34%, and 42.34%, respectively).

### Effect of alternative splicing on nuclear localization

**Figure 7:**
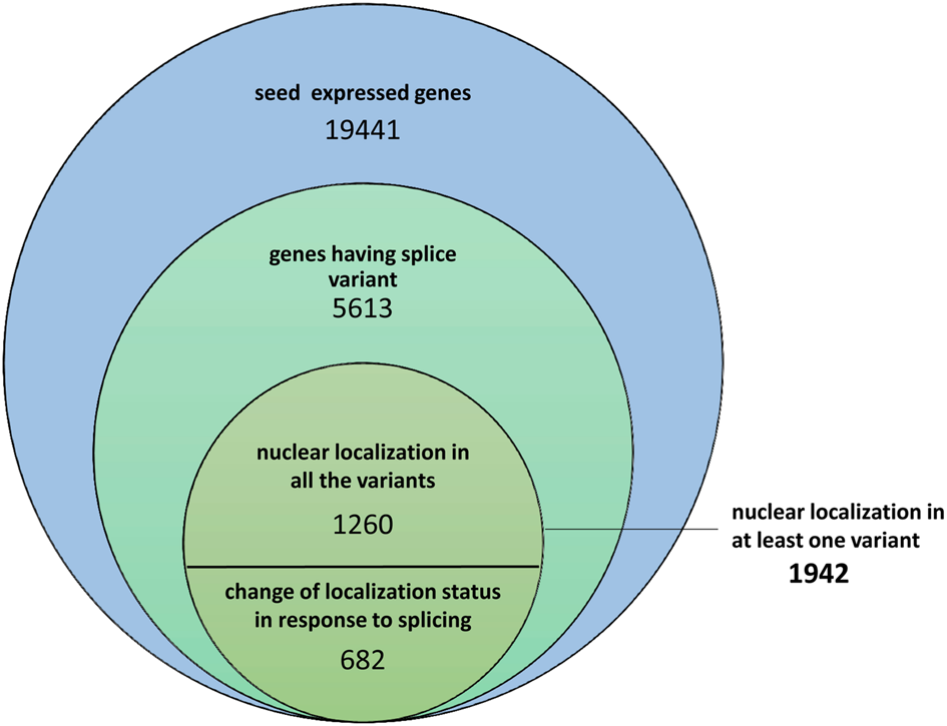
Analysis of alternative splicing and its effect on nuclear subcellular localization. The innermost circle represents the genes that exhibit alternate splicing, and at least one of the protein products is predicted to localize to the nucleus. For 1260 genes, all the variant proteins are predicted to localize to the nucleus, but for 682 genes, variable subcellular localizations are predicted for their products of alternate splicing.

Alternative splicing is a mode of formation of more than one transcript from a gene. Earlier reports have shown that products of splice variants may be localized differentially. We analyzed the presence of splice variants, as reported in the RGAP database. In rice, 6459 genomic loci showed evidence for splice variants. Of these, 5612 genes with splicing alternatives were expressed during seed development of rice. We checked the localization prediction of proteins coded by all splice forms of these genes. It was observed that 1942 genes showed nuclear localization of at least one protein translated from their variably spliced mRNA. In 1260 genes, all splicing forms encoded for nuclear-localized proteins, whereas for 682 genes, splicing resulted in changes in the protein localization. Thus, splicing is also a regulatory factor for the nuclear localization of proteins in seed development in rice (Fig. 7).

### Rice seed nuclear protein database (RSNP-DB)

**Figure 8:**
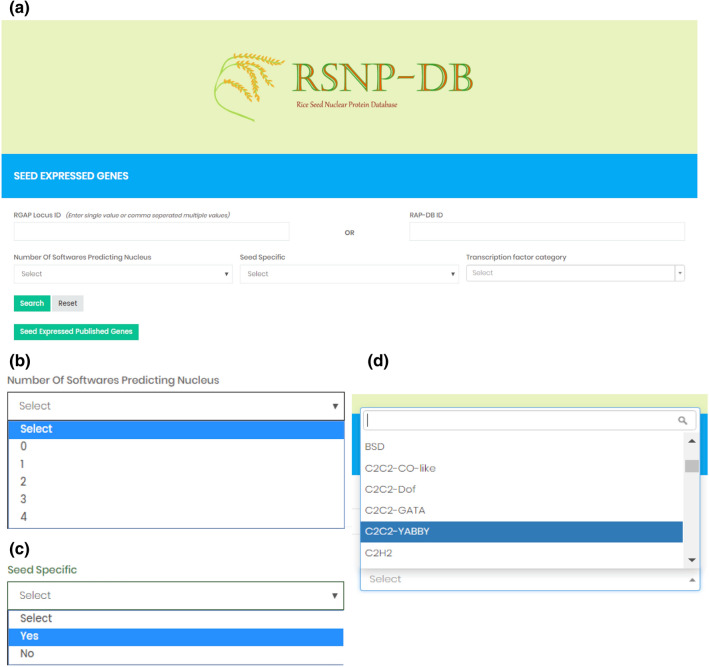
Screenshots of the rice seed nuclear protein database (RSNP-DB). Homepage of RSNP-DB showing various search functions (**a**). Expanded drop down menus for category type, seed specificity and transcription factor category are given in (**b**–**d**), respectively.

We combined all the information in the form of a database named Rice Seed Nuclear Protein Database (RSNP-DB, https://pmb.du.ac.in/rsnpdb). The web interface of RSNP-DB was designed to include the following components: search, information and expression profile for seed-expressed genes. Non-seed-expressed genes are unavailable in the database. The home page integrates the search function for users to access the data stored at RSNP-DB (Fig. 8a). The database can be searched for any RGAP or RAP-DB locus ID (single as well as in batches). The genes can also be searched using the five categories based on the number of softwares predicting the encoded protein to be nuclear (Fig. 8b). Further, transcription factor categories can be retrieved by keywords available in the dropdown menu (Fig. 8d). Genes that have a seed-specific expression are also marked, and the searches can be refined (Fig. 8c). Since RSNP-DB has an interactive user search, the genes can be searched simultaneously for different categories in an additive manner. For example, the ‘MADS BOX transcription factor’ that shows specific expression in seed development having ‘3’ softwares predicting as nuclear protein can be retrieved. In summary, the search option allows users to perform a variety of data retrieval from RSNP-DB.

The search result opens a LOCUS detail page, wherein information for the gene accession is available. Both RGAP IDs and RAP-DB IDs are hyperlinked to their respective sites. Herein, the predicted subcellular localization of each protein by WoLF-PSORT, CELLO, YLoc, and NucPred is available with their respective scores. The information about experimental evidence for nuclear localization of proteins is also included in the database with reference to the published paper in the form of PubMed ID (PMID) linked to NCBI (wherever available) and a link to the paper webpage. The type of cells used for the localization experiment and experimental result are documented for the published genes. Those genes whose localizations are validated by us, the confocal image of YFP fused protein in tobacco leaf epidermal cells is available for localization evidence. A link to all published cloned genes is present on the home page. Details of protein sequence analysis of respective loci are available with the number of PAT4, PAT7, and bipartite signals present in the protein. The protein sequence is also provided. GO annotations with different GO categories and their respective GO terms are presented for the loci. The presence or absence of the alternative splice forms is also marked for all the seed-expressed genes in the database.

Each gene is attached to its expression profile from different experimental sets of microarray data. The expression profiles can be seen in a different page wherein transcript abundance profile of the gene is available for vegetative stages, reproductive development (panicle development, anther development, and seed development) and abiotic stress treatments. The profiles can be seen as bar graphs in terms of normalized expression values. Thus RSNP-DB is a comprehensive compilation of seed nuclear proteome with user-friendly searchability.

## Discussion

The seed is the organ of commercial importance in rice. Its quality and quantity have direct implications on the market value of the crop. Understanding the molecular regulation of seed development is essential to identify tools to bioengineer various seed traits. Nuclear proteins have primarily regulatory functions ranging from epigenetic regulation to transcriptional regulation of genes. In this study, we have seen that those transcripts expressed during seed development showed almost half of their translated products to be nuclear-localized by at least one subcellular-localization prediction tool. Of the 9892 predicted nuclear proteins, the confidence of their actual occurrence in the nucleus increased with more number of softwares predicting a protein to be nuclear. Most computational subcellular prediction tools perform differentially, based on different protein characters, training datasets, training strategies, and algorithms. Combing multiple computational platforms negates the inconsistencies amongst them and supports the confidence of a prediction. This confidence is further strengthened by observing its functional composition. GO enrichment analysis showed a steep elevation from category 2 onwards, in all the terms corresponding to nuclear function (Fig. 2). Also, the bulk of the transcription factor component of seed-expressed proteins that are anticipated to be nuclear is picked up in categories 2, 3, and 4 (Fig. 1).

The presence of NLS and NES is an indication that a protein has spent some time of its life in the nucleus. Proteins encoded by 23.49% of seed-expressed genes carried an NES that underlined that seed nuclear proteins are highly dynamic, and most of them shuttle between the nucleus and cytosol depending upon various factors. It has been predicted that around 35% of all proteins show multiple subcellular localizations^49^. However, nuclear trafficking is regulated at various levels in plants^4^. Many proteins enter the nucleus in the form of a complex. Such mechanisms are called piggy-back import of proteins. All the proteins of the complex need not have the NLS, rather one NLS on any one protein of the complex is enough for the movement. Likewise, the export of protein complexes also does not require NES on every protein. Further, many non-conventional NLSs are reported^2^. Non-classical PY-NLS involved in the nuclear import of protein via karyopherin-β are poorly characterized and difficult to predict due to their multivalent nature^50^. Additionally, a large number of nuclear proteins follow non-conventional nuclear import^51^. Thus, owing to high variabilities in nuclear trafficking mechanisms, only sequence-based prediction cannot identify all nuclear proteins. Finding homologies with known nuclear-localized proteins can be a way to find nuclear proteins. Our results showed that the CELLO prediction tool performed best to predict the validated nuclear proteins. Interestingly, CELLO does not use NLS to predict nuclear proteins; rather, it uses sequences of proteins across genera of known localizations to find closely similar proteins to predict localization^42^. Thus, sequence similarity will take care of the mode of nuclear import in non-conventional mechanisms of import.

Analysis of experimental evidence for localization gave deep insights into the mechanism of nuclear transport. Many examples showed the stress-regulation of nuclear movement. For example, *OsDLK1* (LOC_Os07g01490) that encodes a class-XIV kinesin enters the nucleus only in response to cold^52^. Other examples included the homo/hetero protein complex formation as a means of nuclear localization regulation. For instance, OsMADS29 (LOC_Os02g07430) moves to the nucleus in the dimeric form, whereas the un-associated monomer stays in the cytoplasm^53^. We observed that certain proteins that are very well predicted to be nuclear still do not show experimental evidence for nuclear localization. There could be many reasons for this variation from the prediction. Most of the experimental evidence employs protein fusion with a fluorescent tag that is transiently or stably transformed in either the same or orthologous plant system. Such experiments may miss the right conditions required for the movement of the protein in the nucleus. The reasons could include the absence of the interacting protein, environmental cues, signaling molecule, tissue-specific factors, etc. Hormonal regulation is one crucial factor for nuclear compartmentalization and some examples are worth mentioning. SLENDER RICE1 (SLR1, LOC_Os03g49990) is nuclear predicted by 3 prediction softwares. It localizes in the nucleus in normal untreated conditions, but on the application of GA3, the protein disappears from the nucleus^54^. Likewise, OsJAZ1 (LOC_Os04g55920) is predicted to be nuclear by two prediction softwares, but experimental evidence showed that it stays exclusively in the cytoplasm in the absence of exogenous methyl jasmonate (MeJA). However, the protein showed nuclear localization on treatment with 100 μM MeJA, indicating that it can function as a nuclear protein in the presence of JA signaling^55^.

On the other hand, some proteins that are not predicted by any software as nuclear are experimentally proven to be nuclear-localized. For example, OsGLYI-8 (LOC_Os05g22970) is not predicted by any software to be nuclear, but its localization evidence clearly showed a precise localization in the nucleus. Standard criteria for detection of NLS could not identify any NLS in the protein, but on relaxing the stringency, a very weak NLS was identified that was responsible for nuclear localization because its deletion resulted in cytoplasmic localization of the protein^56^. Thus, the possibility of missing weak or non-canonical NLSs can reduce the identification of actual nuclear proteins. There are at least six non-canonical NLSs identified^57^. Ribosomal proteins and proteins involved in ribosomal biogenesis are classes of proteins that were poorly represented in categories 2, 3, and 4 but were enriched in category 1 and non-nuclear category. Ribosomal proteins are translated from their mRNA in the cytoplasm; however, they move to the nucleolus in the nucleus for their assembly with the newly synthesized rRNA. Thereafter, they are exported back to the cytoplasm for protein translation function^58^. Since they spend the majority of their time in the cytoplasm, their prediction score for cytoplasm is higher than the nucleus for most of the prediction tools. We have picked the organellar localization of the highest score for WoLF-PSORT and CELLO; thus, most ribosomal proteins appeared in low confidence categories.

Alternative splicing can also be a reason for the deviation of prediction from actual localization^59^. Since the sequence of the longest form of proteins was used to predict subcellular localization, we may have missed the alternate localization of variable splice forms that may be functional at a specific stage/condition of development. Thus, we separately checked the localization of all protein variants of the alternative splicing by WoLF-PSORT and found 143 proteins called non-nuclear for the longest form that may have nuclear-localized forms in other variants.

Nuclear proteome, though are the direct evidence of nuclear resident proteins; their isolation and purity are a major concern, especially in seed tissue wherein contamination of abundant seed storage proteins is inevitable. Secondly, the number of proteins identified in all the nuclear proteome studies is much less than the actual predicted nuclear proteins with evidence of transcript. Thirdly, nuclear abundant proteins like ribosomes and histones are over-represented in most proteome studies with poor representation of other low abundance nuclear factors, especially transcription factors^22^. Chromatin-remodeling proteins, including histone binding proteins and histone-modifying proteins, were also well represented in the nuclear proteome studies. Interestingly chromatin remodeler proteins are also abundant in the nucleus^60^. Thus, nuclear proteomes, as of now, reveal only a limited picture of nuclear protein composition. Therefore, a prediction methodology adopting all possibilities of nuclear transport must be used.

## Conclusions

The ability to predict a whole set of nuclear-localized proteins functional in seeds during the grain filling process, without actual experimentation, can help in recognizing a likely molecular network of nuclear interacting proteins that governs the genes responsible for the quality of grains. This nuclear network can act as a toolbox to tweak candidate regulators in a way to enhance grain quality and productivity. RSNP-DB is a database of all seed-expressed genes. This list is the master list wherein the term nuclear protein is searchable. One can simultaneously and interactively procure the information of the nuclear localization confidence, protein sequence features and transcript expression. The availability of information on cloned genes and experimental evidence adds to the comprehensiveness of the database.

## Methods

### In silico analysis

Using our previous microarray expression-data, we identified a list of seed-expressed genes^25, 43^. Briefly, seed development was divided into five temporal stages based on the days after pollination. Microarray data were obtained for three biological replicates of each stage. Application of MAS5 algorithm with default parameters identified expressed proteins with a cut off of at least 66% present call in the triplicate dataset. A MAS5 present call in at least one stage of the seed development was picked up to a list of seed-expressed probeset identifiers (Ids). After converting the microarray probeset Ids into RGAP version 7 locus IDs and removal of redundancy, a final number of 19,441 seed-expressed non-TE genes were obtained. The protein sequences of seed-expressed genes were downloaded from RGAP (https://rice.plantbiology.msu.edu/)^61^. They were used to predict localization using WoLF-PSORT (https://wolfpsort.hgc.jp/), YLoc (https://abi-services.informatik.uni-tuebingen.de/yloc/webloc.cgi), CELLO (https://cello.life.nctu.edu.tw/), and NucPred (https://nucpred.bioinfo.se/nucpred/) online tools. For NucPred prediction, the score above 0.8 was taken as nuclear-localized. For WoLF-PSORT and CELLO, subcellular localization of the highest score was picked up. For YLoc, subcellular localization with the highest probability score was picked up along with its confidence value. All GO terms available for the seed-expressed genes were downloaded from RGAP. GO enrichment analysis was done using the AgriGO V2.0 tool (https://systemsbiology.cau.edu.cn/agriGOv2/)^62^. TF categorization was done according to PlantTFDB (https://planttfdb.cbi.pku.edu.cn/)^63^. For the identification of alternative splice sites, protein sequences of all gene models were downloaded from RGAP and were predicted using WoLF-PSORT. NLS analysis was done using PSORT-II, and NES analysis was done using the NETNES1.1 database (https://www.cbs.dtu.dk/services/NetNES/)^48^. NES scores were identified as sum of NES score of all amino acids of the proteins. Published names of the functionally characterized genes were obtained from funRiceGenes (https://funricegenes.github.io/). The genes were searched manually for the available publications using the published name as keywords. All the locus IDs were crosschecked. Experiments depicting the localization were recorded in the form of the cell type used in the experiment and observed localization organelle. The Pubmed ID (PMID) and journal webpage links were also enlisted.

### Subcellular localization

The selected nuclear proteins were cloned in the pSITE-3CA vector, wherein the *CaMV35S* promoter drove the YFP-fusion proteins^64^. The cloned constructs were mobilized in *Agrobacterium* and infiltrated in *Nicotiana benthamiana*. The leaf epidermal cells were visualized using a confocal microscope (TCS SP5, Leica Microsystems, CMS GMBH, Mannheim, Germany). The bright field and fluorescence images were merged for visualizing the location of expression.

### Database implementation

RSNP-DB was implemented with open source tools MySQL 5.6 on a CentOS Linux (14.04) with an Apache server 2.4.9. The web-based user interface was developed with Hyper Text Markup Language (HTML). The server side scripting language PHP 7.1 was used to communicate with the RSNP-DB database. CSS, JavaScript and JQuery were used for easy search of the RNSP-DB. The user-entered query is sent to a PHP script through an interactive search form. The PHP script sends the queries to the MySQL database, then retrieves and displays the results along with hyperlinks to additional information. Users can retrieve various information by inputting the desired keywords. The system is not case sensitive for search string but sensitive to specific notations/symbols used in its native form.

## Supplementary information

Supplementary Information 1.

Supplementary Information 2.

Supplementary Table S1.

Supplementary Table S2.

Supplementary Table S3.

Supplementary Table S4.
