# Supplementary material for: Analysis of rice nuclear-localized seed-expressed proteins and their database (RSNP-DB)
Source: Sci Rep. 2020 Sep 15;10:15116. doi: 10.1038/s41598-020-70713-8 (PMC7492263; doi:10.1038/s41598-020-70713-8)

Priyanka Deveshwar[1], Shivam Sharma[1], Ankita Prusty[1], Neha Sinha[1], Sajad Majeed Zargar[1,2], Divya Karwal[3], Vishal Parashar[3], Sanjeev Singh[3], Akhilesh Kumar Tyagi[1*]

[1]Interdisciplinary Centre for Plant Genomics and Department of Plant Molecular Biology, University of Delhi, South Campus, New Delhi, India
[2]Proteomics Laboratory, Division of Plant Biotechnology, Sher-e-Kashmir University of Agricultural Sciences & Technology of Kashmir, Shalimar, Srinagar, J&K, India
[3]Institute of Informatics and Communications, University of Delhi, South Campus, New Delhi, India

*Corresponding author, akhilesh@genomeindia.org, 91-11-24115095

Supplementary File 1 : Hierarchical tree graph of enriched gene ontology terms for the four prediction categories in 'Biological Process', 'Molecular function' and 'Cellular Component', GO categories.

Biological Process

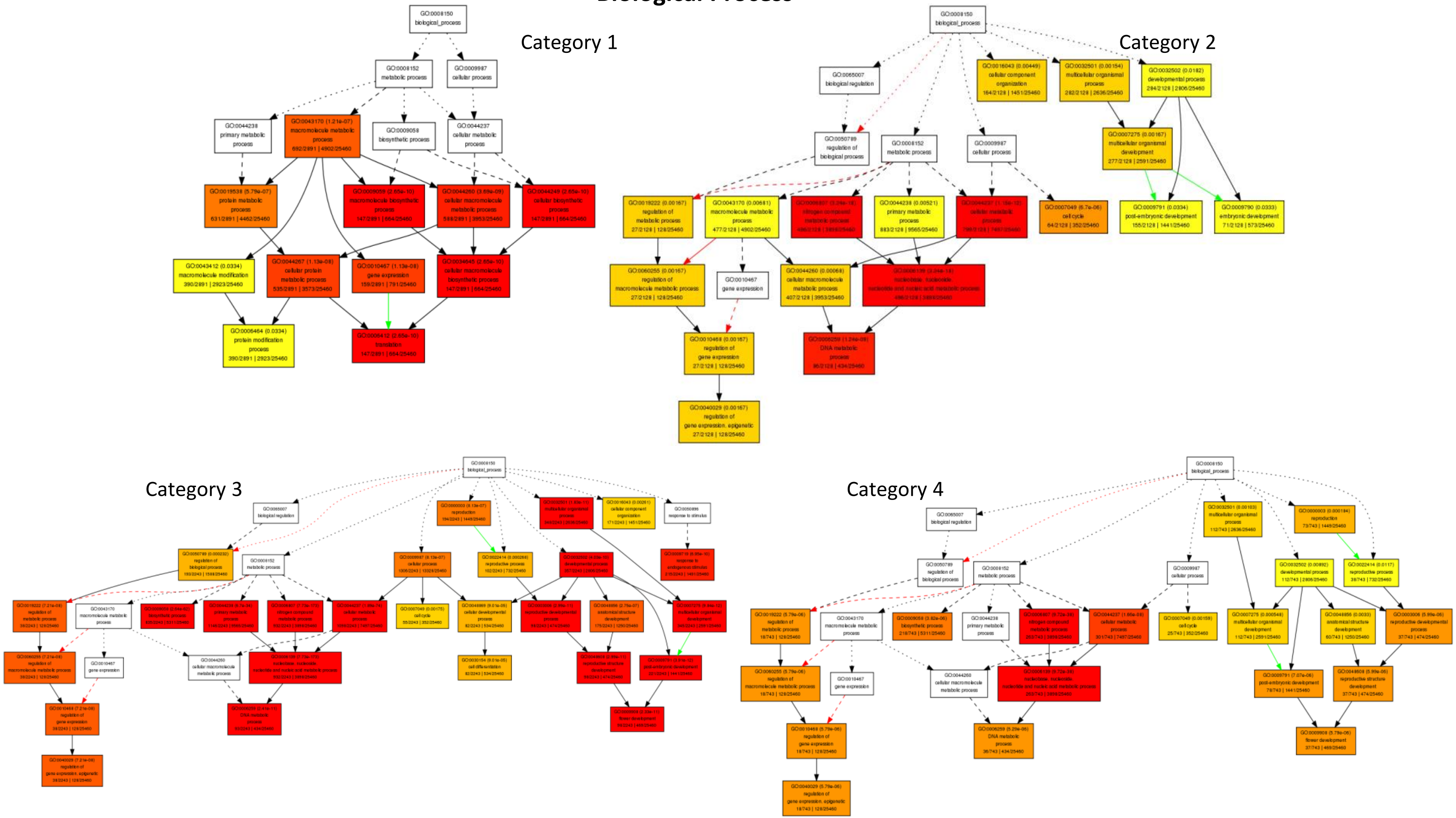

Molecular Function

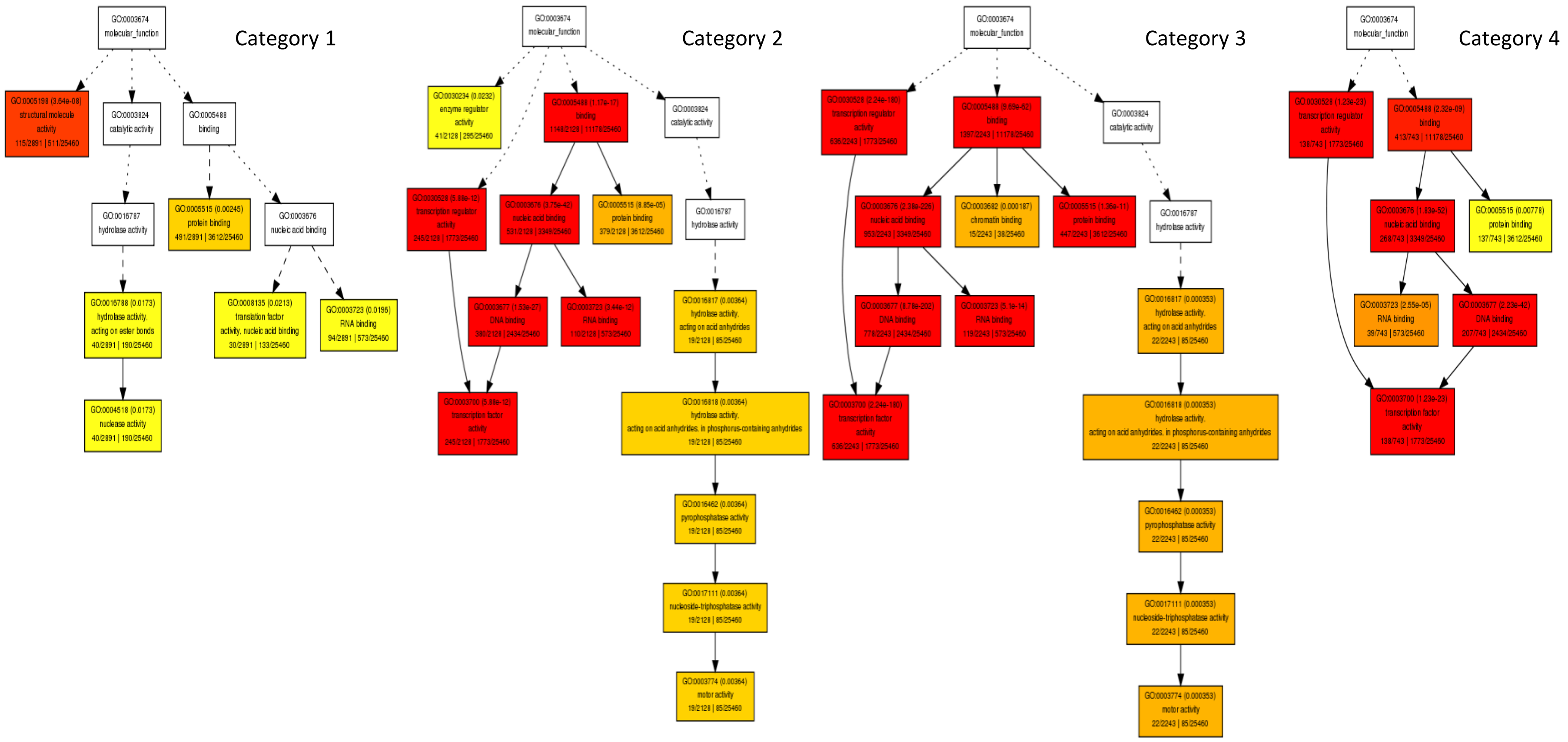

Cellular Component

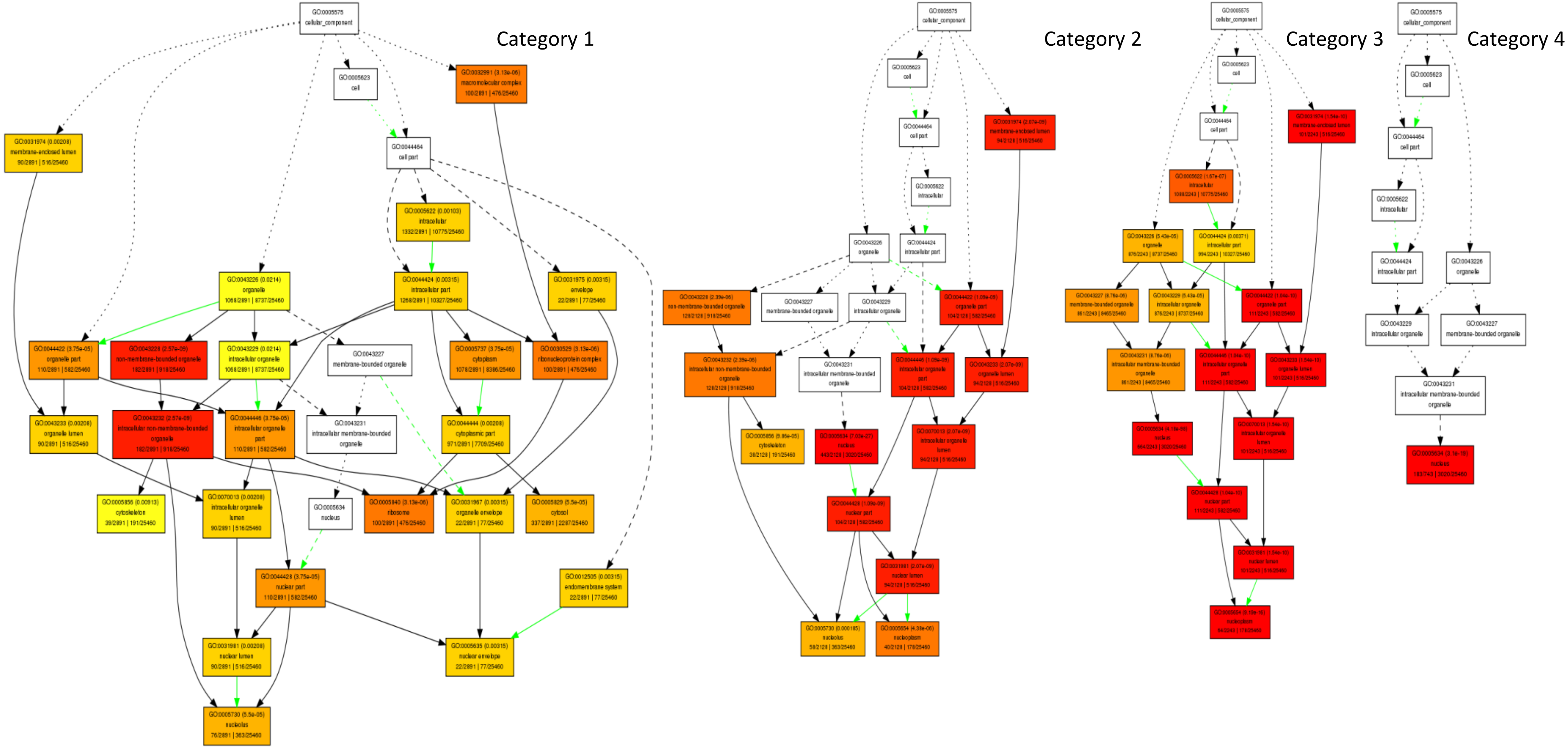

Supplement: Supplementary file 1 — Supplementary Information 1. [file 41598_2020_70713_MOESM1_ESM.pdf]
