# Supplementary material for: Analysis of rice nuclear-localized seed-expressed proteins and their database (RSNP-DB)
Source: Sci Rep. 2020 Sep 15;10:15116. doi: 10.1038/s41598-020-70713-8 (PMC7492263; doi:10.1038/s41598-020-70713-8)
Supplement: Supplementary file 2 — Supplementary Information 2. [file 41598_2020_70713_MOESM2_ESM.pdf]

Analysis of rice nuclear-localized seed-expressed proteins and their database (RSNP-DB)

Priyanka Deveshwar[1], Shivam Sharma[1], Ankita Prusty[1], Neha Sinha[1], Sajad Majeed Zargar[1,2], Divya Karwal[3], Vishal Parashar[3], Sanjeev Singh[3], Akhilesh Kumar Tyagi[1*]

[1]Interdisciplinary Centre for Plant Genomics and Department of Plant Molecular Biology, University of Delhi, South Campus, New Delhi, India
[2]Proteomics Laboratory, Division of Plant Biotechnology, Sher-e-Kashmir University of Agricultural Sciences & Technology of Kashmir, Shalimar, Srinagar, J&K, India
[3]Institute of Informatics and Communications, University of Delhi, South Campus, New Delhi, India
*Corresponding author, akhilesh@genomeindia.org, 91-11-24115095

Supplementary File 2: **Subcellular localization of selected candidate proteins fused with YFP in the leaf epidermal cells of *Nicotiana benthamiana*.** The fused proteins were mobilized in *Agrobacterium* and infiltrated in the leaves. Scale bar = 50 µm.

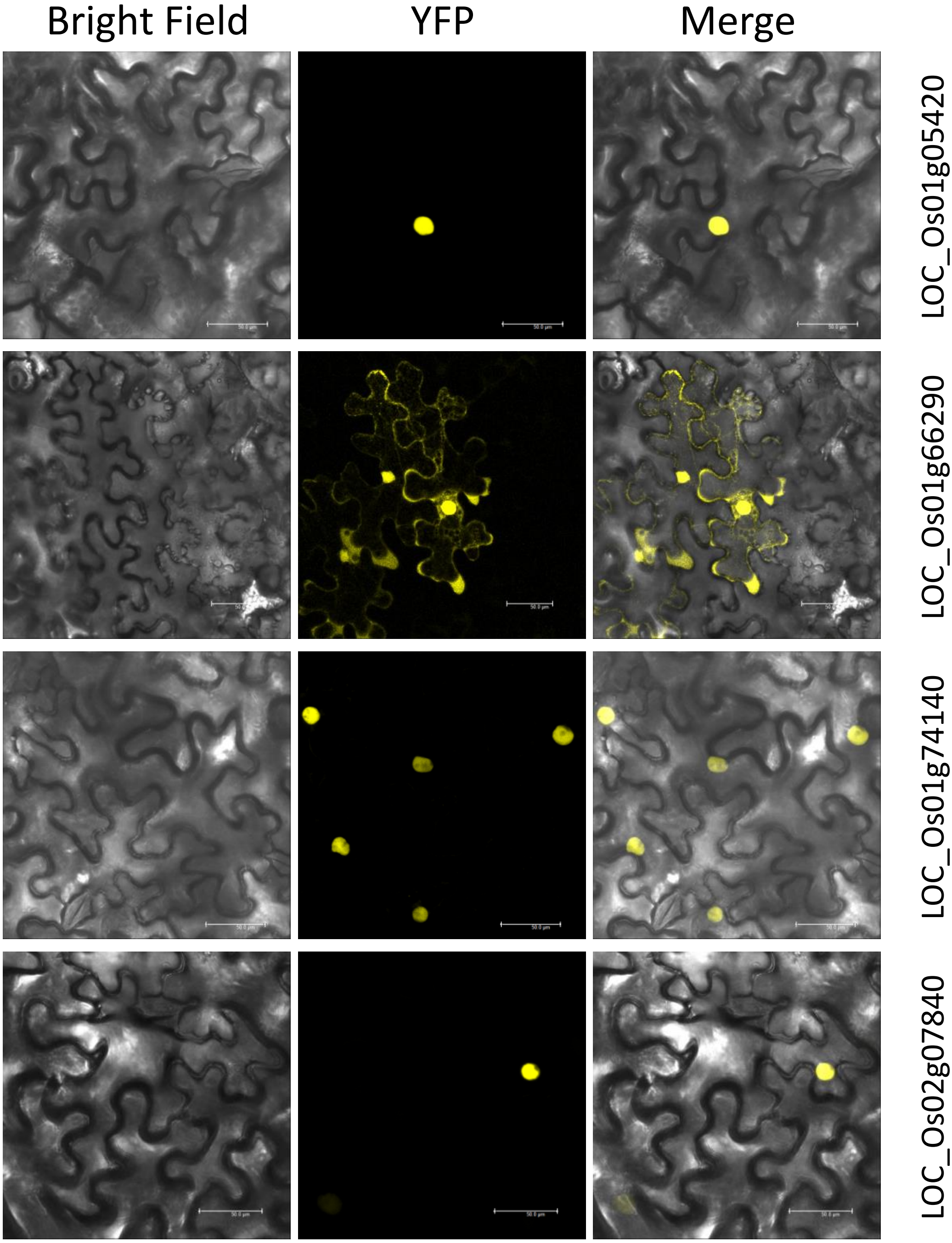

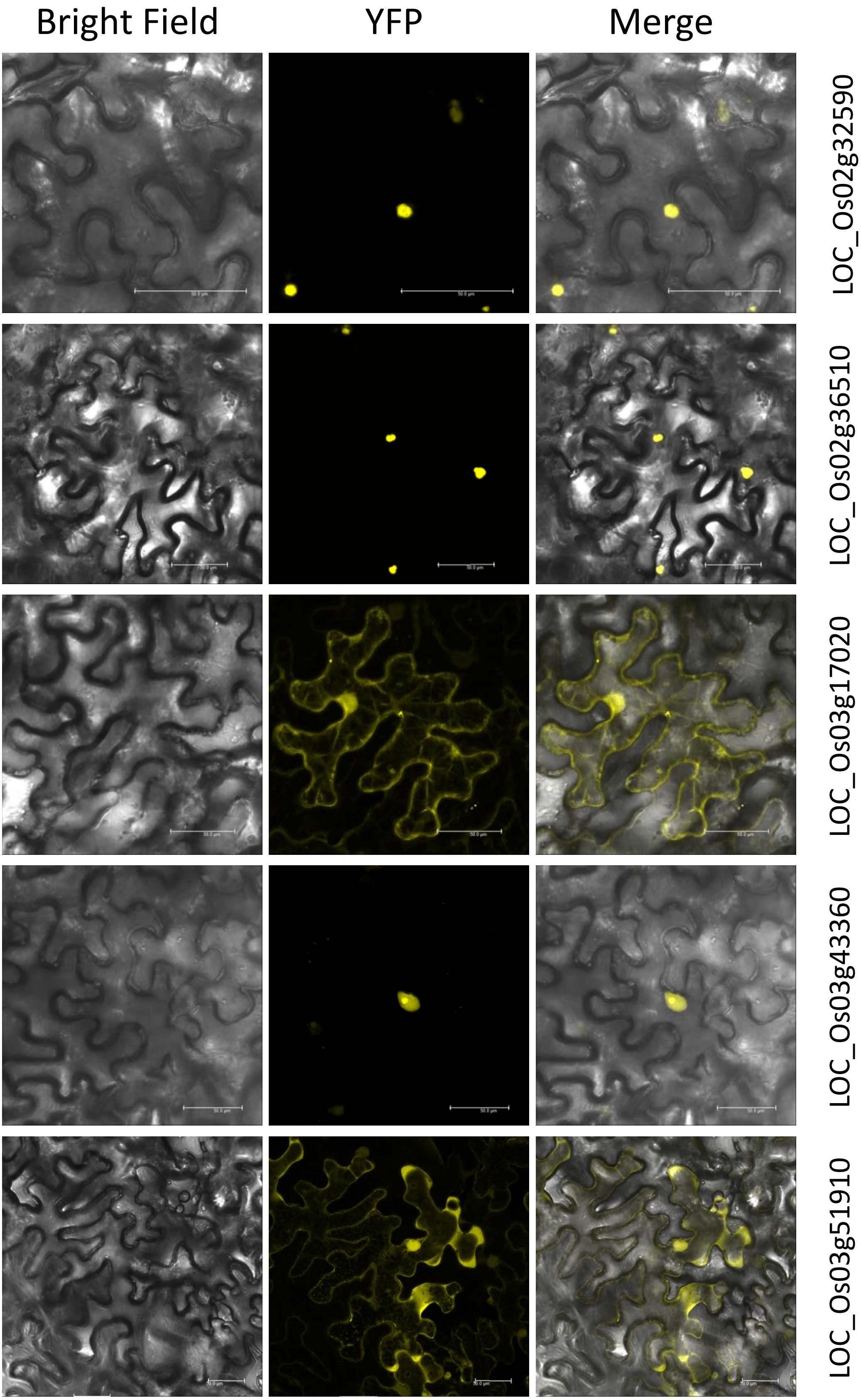

Bright Field
YFP
Merge
LOC_Os02g32590
LOC_Os02g36510
LOC_Os03g17020
LOC_Os03g43360
LOC_Os03g51910

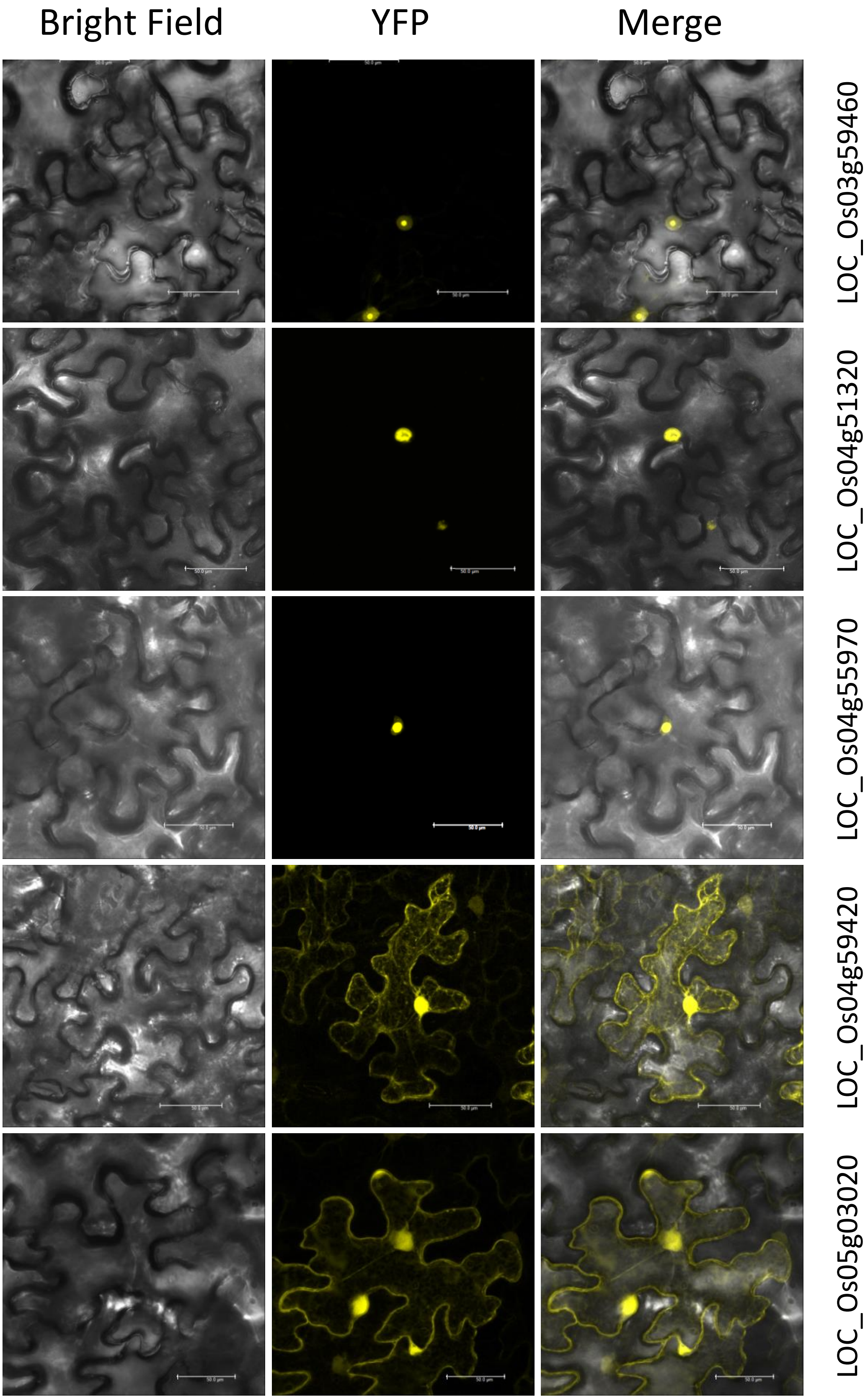
Bright Field
YFP
Merge
LOC_Os03g59460
LOC_Os04g51320
LOC_Os04g55970
LOC_Os04g59420
LOC_Os05g03020

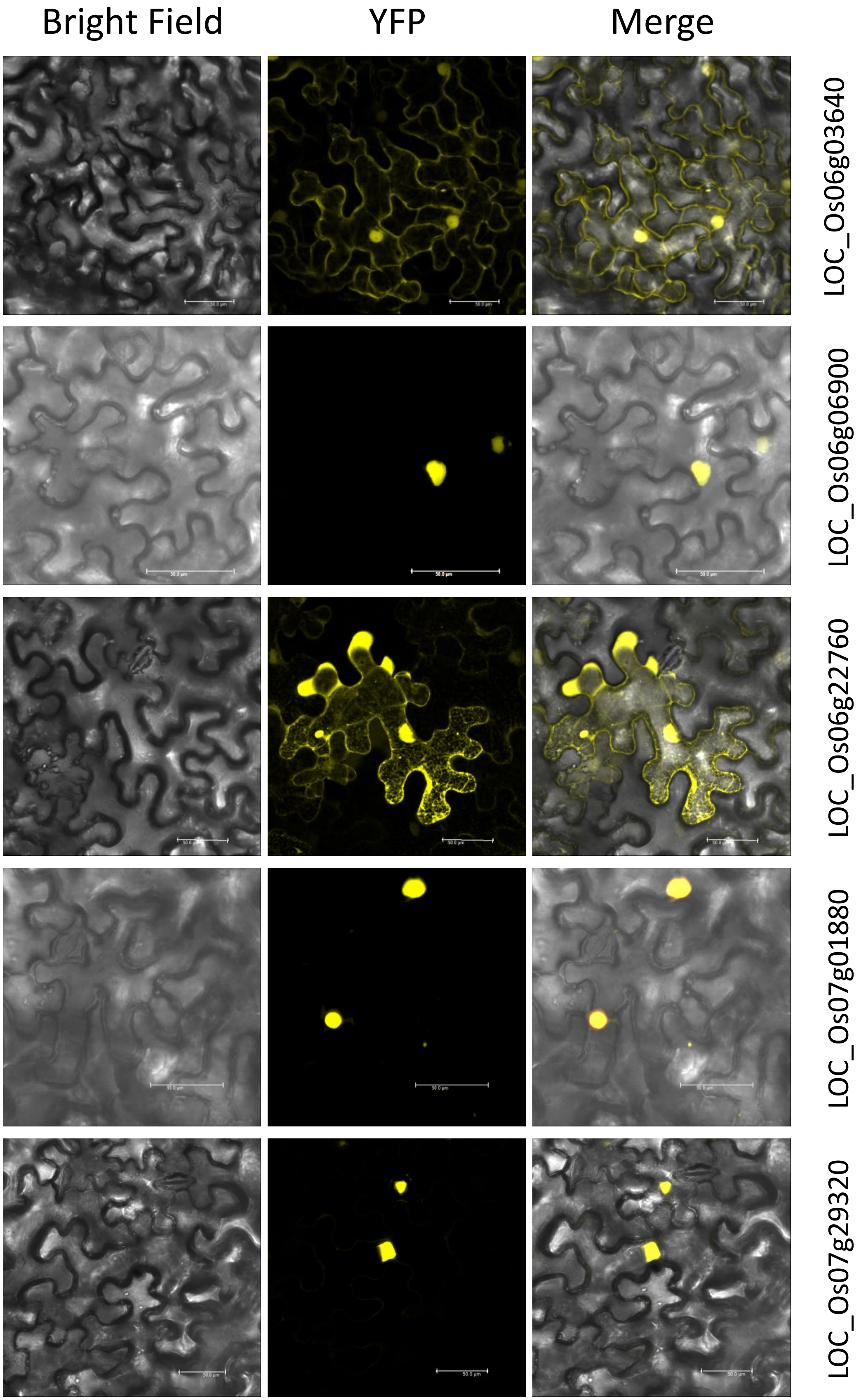

Bright Field
YFP
Merge
LOC_Os06g03640
LOC_Os06g06900
LOC_Os06g22760
LOC_Os07g01880
LOC_Os07g29320

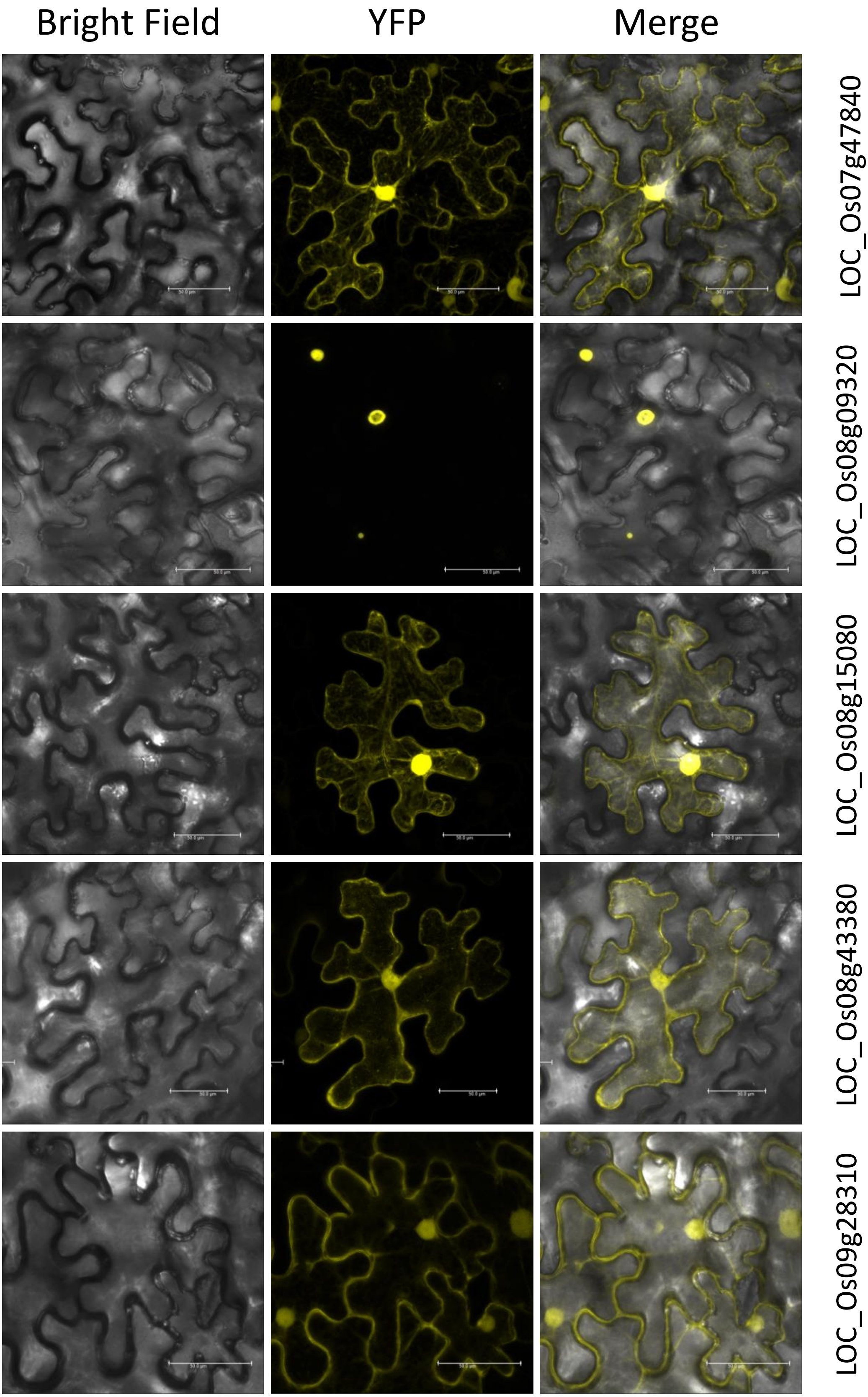
Bright Field
YFP
Merge
LOC_Os07g47840
LOC_Os08g09320
LOC_Os08g15080
LOC_Os08g43380
LOC_Os09g28310

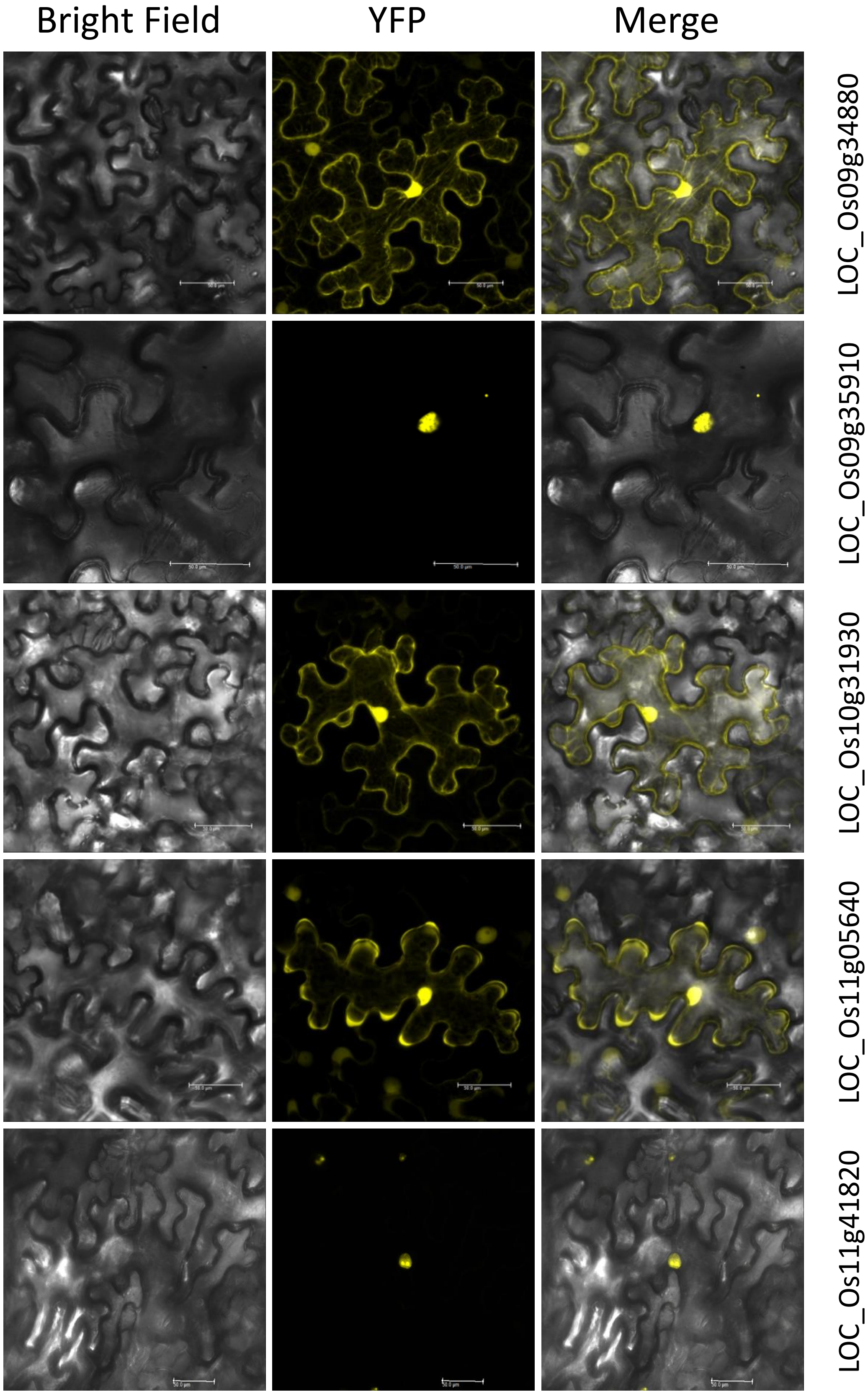
Bright Field
YFP
Merge
LOC_Os09g34880
LOC_Os09g35910
LOC_Os10g31930
LOC_Os11g05640
LOC_Os11g41820

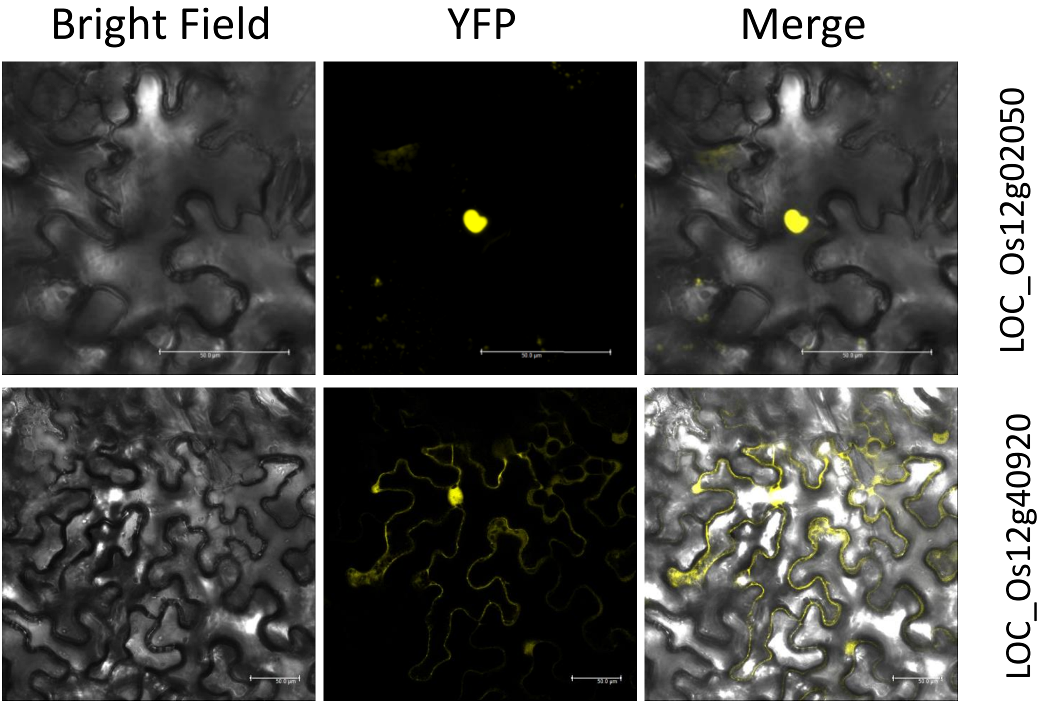

Bright Field
YFP
Merge
LOC_Os12g02050
LOC_Os12g40920
